# Advancing collaborations in health research and clinical trials in Sub-Saharan Africa: development and implementation of a biostatistical collaboration module in the Masters in Biostatistics Program at Stellenbosch University

**DOI:** 10.1186/s13063-021-05427-x

**Published:** 2021-07-22

**Authors:** Tonya M. Esterhuizen, Guowei Li, Taryn Young, Jie Zeng, Rhoderick Machekano, Lehana Thabane

**Affiliations:** 1 Division of Epidemiology and Biostatistics, Department of Global Health, Stellenbosch University, Cape Town, South Africa; 2 Center for Clinical Epidemiology and Methodology, Guangdong Second Provincial General Hospital, Guangzhou City, Guangdong Province China; 3 Department of Health Research Methods, Evidence, and Impact, McMaster University, H325- 50 Charlton Avenue E, Hamilton, ON L8N 4A6 Canada; 4 Research Department, Elizabeth Glaser Pediatric AIDS Foundation, Washington DC, USA; 5 Biostatistics Unit, St Joseph’s Healthcare Hamilton, Hamilton, ON Canada; 6 Departments of Pediatrics and Anesthesia, McMaster University, Hamilton, ON Canada

**Keywords:** Collaboration, Experiential learning, Multidisciplinary, Mentorship, Sub-Saharan Africa

## Abstract

**Background:**

Sub-Saharan Africa continues to carry a high burden of communicable diseases such as TB and HIV and non-communicable diseases such as hypertension and other cardiovascular conditions. Although investment in research has led to advances in improvements in outcomes, a lot still remains to be done to build research capacity in health. Like many other regions in the world, Sub-Saharan Africa suffers from a critical shortage of biostatisticians and clinical trial methodologists.

**Methods:**

Funded through a Fogarty Global Health Training Program grant, the Faculty of Medicine and Health Sciences at Stellenbosch University in South Africa established a new Masters Program in Biostatistics which was launched in January 2017. In this paper, we describe the development of a biostatistical and clinical trials collaboration Module, adapted from a similar course offered in the Health Research Methodology program at McMaster University.

**Discussion:**

Guided by three core principles (experiential learning; multi-/inter-disciplinary approach; and formal mentorship), the Module aims to advance biostatistical collaboration skills of the trainees by facilitating learning in how to systematically apply fundamental statistical and trial methodological knowledge in practice while strengthening some soft skills which are necessary for effective collaborations with other healthcare researchers to solve health problems. We also share some preliminary findings from the first four cohorts that took the Module in January–November 2018 to 2021. We expect that this Module can provide an example of how to improve biostatistical and clinical trial collaborations and accelerate research capacity building in low-resource settings.

**Funding source:**

Fogarty International Center of the National Institutes of Health.

## Introduction

It has been recognized as far back as 1957 that, while recent graduates who specialize in biostatistics are usually adequately equipped to do biostatistical research, they are in fact inadequately prepared to practice as biostatisticians or clinical trial methodologists [1]. Biostatisticians or methodologists working within an academic environment are generally involved in applied research by collaborating with multidisciplinary teams including epidemiologists, healthcare professionals, students from a variety of health sciences programs, and health economists, among others. The challenges in working with such diverse teams consist of dealing with various human elements, addressing a wide range of research problems or disciplines, and working with researchers with minimal statistical background [2]. Additionally, biostatisticians or methodologists themselves might come from a diversity of educational and cultural backgrounds [3]. Moreover, while it has been recognized that both short-term (i.e., consultation) and long-term (i.e., collaboration) statistical and methodological activities require sophisticated non-statistical skills [4], unfortunately, the skills needed are not usually taught in biostatistics or clinical trials postgraduate programs [5, 6]. All these aspects can impact the efficiency and effectiveness of communication between biostatisticians or clinical trial methodologists and other clinical researchers.

Sub-Saharan Africa continues to carry a high burden of communicable diseases such as tuberculosis and HIV, and non-communicable diseases such as hypertension and other cardiovascular conditions, as well as injuries [7, 8]. Although investment in conducting relevant research, and in increasing the uptake of research into healthcare policy and practice [9], has led to advances and improvements in health outcomes, a lot still remains to be done to build research and knowledge translation capacity in health [10]. Like many other regions in the world, in Sub-Saharan Africa, biostatisticians and clinical trial methodologists remain a scarce resource, which leads to an over-reliance on input from biostatisticians sourced from high-income countries or the pharmaceutical industry for writing research grants, performing advanced data analyses, publishing in high-profile journals, teaching biostatistics courses, and training postgraduate students [11]. To mitigate this issue, the Masters in Biostatistics Program (subsequently called “the Program”) at Stellenbosch University was started in 2017 after a meeting of Sub Saharan African Biostatisticians in 2014 that identified biostatistical training as a specific need in the region [12]. At the meeting, the demand to improve the trainees’ non-statistical (often called soft) skills was also highlighted, where these skills include effective collaboration and consulting skills, life-long learning, efficient communications, and leadership training, among others [12]. To enhance the capacity building of both statistical and soft skills, Stellenbosch University introduced a 12-credit Biostatistical Consulting and Collaboration Module (subsequently called “the Module”) as part of the Program running in the second year from January to November. It was offered in 2018 in collaboration with a senior biostatistician who had vast experience in developing and teaching similar courses at McMaster University [5] and an in-house module facilitator with 15-year experience in biostatistical consulting and collaborating in Sub-Saharan African university environments. In this article, we briefly introduce the outline of the Module and show some preliminary findings from the Module starting in 2018. It is expected that the Module can help improve the biostatistical collaboration and accelerate research capacity building for students in Sub-Saharan Africa.

## Outline of the Module—principles, skills, lectures and practice, linkage with internship, and assessment

**Fig. 1:**
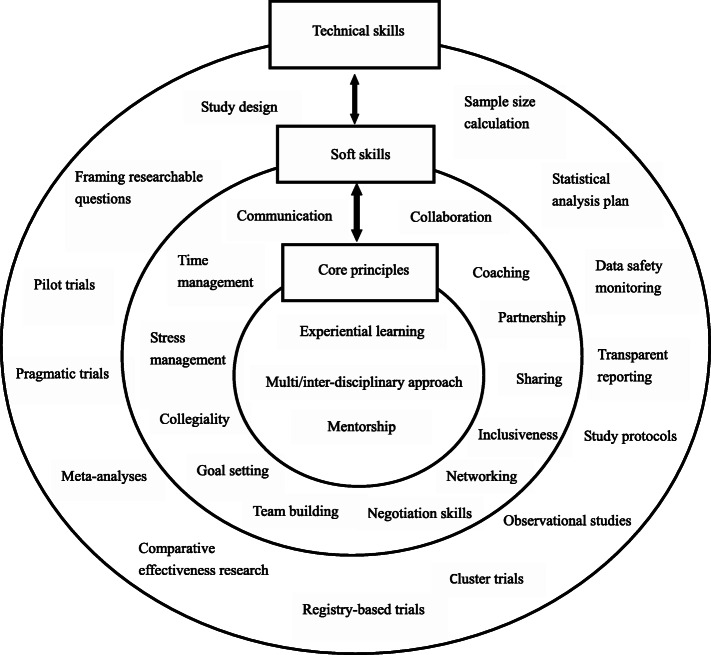
Core principles and fundamental soft and technical skills for the Module

Figure 1 shows the overview of the Module. The Module is guided by three principles and covers both the fundamental technical and soft skills for the biostatistical and methodological collaborations. All the three parts are interactively implemented during the Module activities.

### Guiding core principles

We adopted the existing successful module at McMaster University in the Program [5]. The Module includes three guiding core principles: (1) embracing experiential learning, (2) using a multidisciplinary approach in collaborations, and (3) identifying a formal mentor (including “mentorship-beyond-borders”) (Fig. 1). Students trained with the Module at McMaster University generally proactively get involved in various research groups that engage local, national, and global communities in identifying problems and finding solutions. Such research environments provide our students experiential learning and shared opportunities that help them locate research needs and improve their collaborations. A second principle using a multidisciplinary approach is recommended because both efforts to generate primary clinical research evidence and developments of clinical guidelines leading to improvements in health require multidisciplinary backgrounds and clinical research network [13]. Embracing a multidisciplinary environment can advance biostatistical collaborations to yield high-quality outputs in an effective fashion. Another principle is related to mentorship. Evidence has shown that a strong mentorship can help achieve great career satisfaction, accelerate research productivity, expand research networks, offer more opportunities, advance academic promotion, and enhance the acquisition of important career skills, to mention a few [14–18]. Therefore, our students trained in the Module are always encouraged to identify an experienced mentor as an important step in their career.

### Technical and soft skills for collaboration

Figure 1 also displays the technical and soft skills needed for effective biostatistical collaborations in health research. Being able to apply statistical knowledge in the design, conduct, analysis, reporting, and translation of studies is the fundamental requirement for a qualified biostatistician in collaboration with non-statisticians [3, 19]. In the Module, we seek to cover some most common and relevant aspects in real-life collaborations, including pragmatic trials (design), data safety monitoring (conduct), statistical analysis plan (analysis), transparent reporting (reporting), and meta-analyses (translation), among others. The soft skills, on the other hand, play a key role in successful collaborations with researchers from different culture and backgrounds to solve complex problems [19, 20]. We have to emphasize that such soft skills are usually not taught in regular statistics or biostatistics graduate programs, and therefore many graduates have to learn these skills through trial-and-error when they start working [21, 22]. Facilitating learning of our students in soft skills in biostatistical and methodological collaborations is an important element that makes the Module unique and intriguing.

### Lecture topics and practice

Guided by the three principles, the Module, with a total of 120 h learning time, was designed to comprise of 1/3 face-to-face lectures (40 h) and 2/3 practical work (80 h) implemented throughout the module. Table 1 shows the schedules of the learning time assigned for the Module.

**Table 1:** Schedules of the learning time for the Module

	Schedule of the learning time		
**Face-to-face lectures** including 2 one-week-blocks learning time	February (20 h)	April (20 h)	
**Practice work** implemented throughout the Module	Directed self-study (12 h)	Self-directed study (36 h)	Formative assessments (32 h, including two assignments and a presentation)

The objectives of lectures and practice include the following: (1) integrating statistical knowledge gained in the other modules and applying it during biostatistical and methodological consulting sessions with clinical researchers and students, (2) communicating effectively with researchers to gather information required to make a link between research questions and statistical methods by asking relevant questions, (3) providing guidance in study design, sample size calculations and statistical analysis plans for researchers, (4) effectively analyzing data from consultations on research projects within a variety of biomedical fields, (5) discussing results in a clear manner orally and in the form of a written report to researchers, (6) doing research on the relevant statistical methodologies and presenting findings to the audience, and (7) displaying teamwork and leadership in research collaborations. Details on the topics and practice including their contents and key references for the Module are shown in Table 2. For example, the second topic is about the communication skills between a biostatistician and researchers. The topic aims to train students how to raise questions thoughtfully, understand the do’s and don’ts during communications, appreciate the culture and backgrounds of collaborators, make others aware of your statistical and social culture, and present statistical methodology and plans to the non-statistical audiences. Readers can refer to reports from Ehrenberg [23], Zahn and Isenberg [4], and others for further details regarding communication between biostatisticians and researchers.

**Table 2:** Lecture topics and practice, contents, and references for the Module

Topic/practice	Contents	Key references
Biostatistical collaboration in health research	• Understanding the role of a statistician in health research collaborations • Negotiating your role in collaborations • How to balance service versus leadership roles • Dealing with difficult ethical dilemmas	• American Statistical Association. Ethical standards for statistical practice: Report of the ad hoc committee on professional ethics. The American Statistician 1983; 37: 5-6. • Engeman RM, Shumake SA. Animal welfare and the statistical consultant. American Statistician 1983; 47: 229-33. • Hooke R. Getting people to use statistics properly. The American Statistician 1980; 34: 102-7. • American Statistical Association. Ethical Guidelines for Statistical Practice. 1999. http://www.amstat.org/profession/index.cfm?fuseaction=ethicalstatistics
Communication (verbal, written and presentations)	• The art of asking questions in collaborations • The do’s and don’ts of communication in collaborative research • Understanding the clinical environment and culture of your collaborators • Getting your collaborators to understand your statistical and social culture • How to give a technical talk to a non-statistician audience	• Ehrenberg ASC. Writing technical papers or reports. The American Statistician 1982; 36: 326-9. • Zahn DA, Isenberg DJ. Nonstatistical Aspects of Statistical Consulting, The American Statistician 1983; 37: 297-302. • Day RA. How to Write and Publish a Scientific Paper. Oryx: Phoenix, 1994 • Ehrenberg ASC. Rudiments of numeracy. Journal of the Royal Statistical Society 1977; A140: 277-97. • Hoadley RB, Kettenring JR. Communication between statisticians and engineers/physical scientists’, Technometrics 1990; 32: 243-74. • McDonald GC. Communicating with managers. Chance 1988; 1: 42-4.
How to frame researchable questions	• Discussing the importance of framing research questions appropriately • Helping your collaborators to frame researchable questions	• Thabane L, Thomas T, Ye C, Paul J. Posing the research question: not so simple. Canadian Journal of Anesthesia 2009; 56: 71-9 • Rios LP, Ye C, Thabane L. Association between framing of the research question using the PICOT format and reporting quality of randomized controlled trials. BMC Medical Research Methodology 2010: 10: 11 • Sackett DL, Wennberg JE. Choosing the best research design for each question. BMJ. 1997; 315(7123): 1636.
Determinants of a successful career development in collaborative research	• Understanding the importance of key determinants of career development success in collaborative research: mentorship, time-management, stress-management, conflict-resolution, managing meetings, etc. • Understanding why, and how to choose a mentor • Discussing co-authorship with collaborators • How to stay abreast with developments in the field	• Sackett DL. On the determinants of academic success as a clinician-scientist. Clin Invest Med 2001;24:94-100. • Sackett DL. Clinician-trialist rounds: 3. Priority setting for academic success. Clinical Trials. 2011; 8: 235-7. • Oxman AD, Sackett DL. Clinician-trialist rounds: 14. Ways to advance your career by saying ‘no’–part 2: When to say ‘no’, and why. Clinical Trials 2013;10:181-187. • Oxman AD, Sackett DL. Clinician-trialist rounds: 15. Ways to advance your career by saying ‘no’–part 3: how to say ‘no’, nicely. Clinical Trials 2013;10:340-343. • Odueyungbo A, Thabane L. Mentoring in biostatistics: some suggestions for reform. Journal of multidisciplinary healthcare 2012;5:265. • Sambunjak D, Straus SE, Marušić A. Mentoring in academic medicine: a systematic review. JAMA 2006;296:1103-1115.
Writing protocols, sample size justification, statistical analysis plans, book reviews, manuscript and grant reviews.	• Understanding how to review and write statistical sections of study protocols • Understanding how to write a statistical analysis plan (SAP) • Understanding how to write a book review • Understanding how to review manuscripts, protocols, grants, and SAPs	• EQUATOR network. London: the Centre for Statistics in Medicine, NDORMS, University of Oxford; 2016. Available from http://www.equator-network.org/. • Chan AW, Tetzlaff JM, Gøtzsche PC et al. SPIRIT 2013 explanation and elaboration: guidance for protocols of clinical trials. BMJ 2013;346:e7586. • Gamble C, Krishan A, Stocken D et al. Guidelines for the content of statistical analysis plans in clinical trials. JAMA 2017;318:2337-2343. • Thabane L, Ma J, Chu R et al. A tutorial on pilot studies: the what, why and how. BMC medical research methodology 2010;10:1.
Reporting of studies	• Understanding the importance of good reporting in science • Understanding how to report studies of different designs including observational studies, RCTs, pilot trials, pragmatic trials	• Nicholls SG, Langan SM, Benchimol EI, Moher D. Reporting transparency: making the ethical mandate explicit. BMC medicine 2016;14:44. • Li G, Bhatt M, Wang M, Mbuagbaw L, Samaan Z, Thabane L. Enhancing primary reports of randomized controlled trials: Three most common challenges and suggested solutions. Proceedings of the National Academy of Sciences 2018;115:2595-2599. • Li G, Abbade LP, Nwosu I et al. A scoping review of comparisons between abstracts and full reports in primary biomedical research. BMC medical research methodology 2017;17:181. • Li G, Abbade LP, Nwosu I et al. A systematic review of comparisons between protocols or registrations and full reports in primary biomedical research. BMC medical research methodology 2018;18:9. • Glasziou P, Altman DG, Bossuyt P et al. Reducing waste from incomplete or unusable reports of biomedical research. The Lancet 2014;383:267-276. • EQUATOR network. London: the Centre for Statistics in Medicine, NDORMS, University of Oxford; 2016. Available from http://www.equator-network.org/.
Special topics in trials	• How to generate a randomization schedule • Dealing with multiplicity issues in trials • Subgroup analyses in trials • Missing data in trials • Data safety monitoring boards • Common errors in study proposals or protocols	• Haynes BR, Sackett DL, Guyatt GH, Tugwell P. Clinical Epidemiology: How to do clinical practice research (3rd Edtion). Lippincott Williams & Wilkins, 2006 • Li G, Taljaard M, Van den Heuvel ER et al. An introduction to multiplicity issues in clinical trials: the what, why, when and how. International journal of epidemiology 2016;46:746-755. • Sun X, Ioannidis JP, Agoritsas T, Alba AC, Guyatt G. How to use a subgroup analysis: users’ guide to the medical literature. JAMA 2014;311:405-411. • Little RJ, D'agostino R, Cohen ML et al. The prevention and treatment of missing data in clinical trials. NEJM 2012;367:1355-1360. • Morse MA, Califf RM, Sugarman J. Monitoring and ensuring safety during clinical research. JAMA 2001;285:1201-1205.
Video sessions (live consultation sessions with collaborators)	• Understanding the importance of good communication skills in discussing research problems with collaborators	None

### Linkage with internship

As part of the Program, students participate in an internship program during the last three months (from July to September) of their second year of study. Although the internship is not included in the Module’s 120-h learning time, we utilize and link the internship as a supplemental platform of real-life practice for students in this Module. Moreover, we use the internship to help with summative assessment of students’ performances (details below).

The internship placements may occur within research institutions or centers where biostatisticians are employed, and the students will usually organize their own placements according to their specific area of interest. The placement organization provides the student with opportunity to engage with researchers, conduct statistical consultation, and gain experience in communication and teamwork under the supervision of an experienced biostatistician. The learning outcomes are to apply theoretical concepts to day-to-day problems in a workplace environment, provide guidance in study design and statistical analysis plans, work in a collaborative environment, and successfully propose solutions to statistical problems arising from workplace environment. Thus, linkage with the internship is a significant way for students to put into practice what they have learned in the Module.

### Assessment of students’ performances

The assessment of students’ performances for the Module is 50% formative and 50% summative. The key points for assessments consist of students’ ability to translate research questions into statistical questions, assistance with study design, analyses, interpretation and reporting of results, effective communications with non-statisticians, self-learning, teamwork, and leadership. The formative part is composed of a presentation (30% of the overall mark) and two written assignments (20% of the overall mark). For the presentation, students are expected to identify a biostatistical topic of interest and relevance to biomedical researchers and then present for 30 minutes to the audience from the Division of Epidemiology and Biostatistics as well as other interested researchers. Marks are awarded for choice of topic, clarity of presentation, knowledge of the subject, and how questions are handled. The written assignments are designed to cover issues that may or may not have been dealt with in the lectures but that are commonly encountered in real-life biostatistical collaboration. The rationale for the assignment settings is to expose students to the actual scenarios that biostatisticians encounter, encourage them to improve self-learning and critical thinking, and enhance their problem-solving ability. For example, one of the assignments concerns randomization in a large multi-center trial, where the treatment groups were not balanced due to chance after the trial ended. Students are asked to provide their suggested solutions, rationale for the solutions, expected analyses and reporting, and concluding remarks appropriate for future similar issues. In general, there are no explicitly correct or incorrect answers to these open questions; therefore, the assignments are marked as an essay with a rubric awarding marks to how well the student has given evidence to support their answer.

The summative part, assessed after the internship, is entirely composed of a portfolio of evidence of learning (50% of the overall mark). Since this Module is primarily applied, it is difficult to summatively evaluate the skills they have learnt using traditional examination format. A portfolio of evidence is a more valid way to help students to better incorporate the knowledge gained through lectures, readings, assignments, and during the real-life practice of their internship. Students document their learning process in biostatistical collaborations through their workplace placements using reflections, case studies reports, written reports to clients, and videos of consultations. The portfolio is assessed through a rubric that examines the depth of their reflection into the learning process as well as the overall quality of the portfolio.

## Findings from the Module from 2018 to 2021

### Evaluating the Module

For the first intake in 2017, we received eight applications and five met the requirements and enrolled. These five students, three from Zimbabwe and two from South Africa, had backgrounds ranging from statistics to health science. They went on to take the Module in their second year of study from January to November 2018. All students were assigned a mentor during the Module who then became their research assignment supervisor. Subsequently, the interest in the program has grown and we receive more than forty applications annually. A further five students were enrolled in 2018 who went on to take the Module in 2019, of which four were Zimbabwean and one was Namibian. The 2019 intake, which took the Module in 2020, consisted of eight students, two of whom were South Africans, and the remainder from Zimbabwe, Namibia, Kenya, and Ethiopia. Thus far, the program has produced sixteen graduates. The 2020 cohort of students who took the Module in 2021 consisted of seven students of whom three were Ethiopian, two South African, one Kenyan, and one Zimbabwean.

Immediately after each teaching block, the students were asked to rate each of the seven following domains of the Module on a scale of 1 (poor) to 7 (excellent): the Module overall so far; the clarity of the Module objectives; the organization of the Module; the relevance to career, educational goals, and interests; the usefulness of the class discussions; the usefulness of the course materials; and how students’ needs and expectations were met.

Block 1 evaluations for 2018 to 2021 cohorts are shown in Table 3. In 2018, the means of the items ranged from 5.6 to 6.2, indicating students’ satisfaction with the Module. The means of the items increased in 2019, 2020, and 2021, as reflected in Table 3.

**Table 3:** Student evaluation scores for the Module from 2018 to 2021

			The module so far	The clarity of the module objectives	The organization of the module	The relevance to career, educational goals, and interests	The usefulness of class discussions	The usefulness of course materials	How students’ needs and expectations were met
Year	2018	N	5	5	5	5	5	5	4
		Mean (sd)	6.0 (0.7)	5.8 (1.3)	5.6 (1.1)	6.0 (1.2)	6.2 (0.8)	5.6 (1.5)	5.8 (1.3)
	2019	N	4	4	4	4	4	3	4
		Mean (sd)	6.8 (0.5)	6.8 (0.5)	6.3 (1)	7.0 (0)	6.8 (0.5)	6.3 (0.6)	6.5 (0.6)
	2020	N	4	4	4	4	4	4	4
		Mean (sd)	6.5 (0.6)	6.3 (0.5)	6.3 (0.5)	6.5 (0.6)	6.5 (0.6)	6.5 (0.6)	6.3 (0.5)
	2021	N	8	8	8	8	8	8	7
		Mean (sd)	6.6 (0.5)	6.6 (0.5)	6.5 (0.5)	6.7 (0.5)	6.6 (0.5)	6.5 (0.5)	6.6 (0.5)

*sd* standard deviation, *N* sample size

Additionally, students were given three open questions on the evaluation form: (1) What are the best features of the Module; (2) Did you feel that any aspects of the Module were not relevant; and (3) Which aspects of the Module would you change? The 2020 cohort of students were given an extra question on the online format of the course due to the COVID-19 pandemic and subsequent lockdown. In each case they were asked to elaborate on their answers. The responses regarding the best features of the Module were mainly around the lecturer’s passion, vast experience, and in-depth understanding of the subject. The real-life practical aspects were also appreciated. From 2019 onward, the responses to this question were mainly around the usefulness of the collaboration framework and the enjoyment of the role-play sessions. They also indicated a renewed appreciation for collaboration as a career and a taste of what to expect in the real world. In terms of the second question, all but one student felt that all aspects were relevant. The one student who felt differently highlighted that the Module placed too much emphasis on “soft skills” such as time management and stress management, at the expense of more important statistical topics. Suggestions for what to change included the addition of more information on sample size calculations and statistical analysis plans and research protocol development. We have considered all their feedback and are now trying to reach a consensus about how to incorporate appropriate changes into the future Module. Suggestions of what to change in future included having conversations with biostatisticians who work collaboratively in different fields or being able to observe real or simulated collaboration sessions. Responses from the 2020 cohort on the new online format of the offering were generally in favor of the format, with students feeling that not much was lost without face-to-face contact; however, one student acknowledged the challenges of internet connectivity and electricity interruptions which happened intermittently in South Africa.

One of the improvements which were done to the Module since 2018 was to add a structured framework for managing and running statistical collaborations [24]. Further improvements under consideration for future years are methodological research within clinical trials (SWAT) [25], methodological issues around systematic reviews and meta-analysis, and economic analyses in trials [26].

### Evaluating students’ performances

While all students progressed well in the formative assessments, the summative portfolios displayed only surface reflections which were almost “report like.” We realize that proper reflection is a skill that has not been taught to the students and this will be incorporated into the future Module. On the positive side, the students tended to be brutally honest about the course and provided very useful feedback about what worked this year and which aspects needed improvement. Moreover, their documentation of the challenges they faced in the Module (reading, lectures, assignments, presentation, and linkage with internship) was also helpful in knowing where the facilitators can be of more assistance in the future. For example one student wrote “The department should have field visits to assess the progress of the students and know challenges faced by students. Also, students should meet on a monthly basis to present their progress on projects and other reports or problems which they face while on field attachment.” Another wrote “More so, I was not spared from problem consultations where some postgraduate students wanted the statistician to do everything for them. Some supervisor who had little understanding of statistics and epidemiological study design would cause confusion between the consulting activities. However, through communication of the problems encountered to the head and seeking advice from other professionals around, I was able to solve and move on without further friction on how to conduct biostatistical collaborations.”

## Concluding remarks

Realizing the urgent need of statistical or methodological expertise and soft skills in biostatistical and clinical trial collaborations, we developed and implemented a module with the purposes of enhancing students’ capacity building at Stellenbosch University in the Program. The Module tried to advance our students’ both technical and non-technical statistical and methodological competencies, using an interactive approach combining in-class instruction, critical thinking and self-learning, and real-life practice. It not only equipped students for engagement to conduct research but also for engagement and communication to advance the use of research in healthcare decision making. Further improvement of the Module is needed in the future setting. It is our expectation that the Module can provide an example of how to improve the biostatistical and clinical trial collaborations and accelerate research capacity building in low-resource settings.

## Data Availability

No data available.
